# Application of Fullerenes as Photosensitizers for Antimicrobial Photodynamic Inactivation: A Review

**DOI:** 10.3389/fmicb.2022.957698

**Published:** 2022-07-14

**Authors:** Wenjia Hou, Guorui Shi, Songze Wu, Jiayi Mo, Lan Shen, Xiuqiang Zhang, Yabin Zhu

**Affiliations:** ^1^School of Medicine, Ningbo University, Ningbo, China; ^2^Key Laboratory of Marine Materials and Related Technologies, Zhejiang Key Laboratory of Marine Materials and Protective Technologies, Ningbo Institute of Materials Technology and Engineering, Chinese Academy of Sciences, Ningbo, China; ^3^Ningbo Key Laboratory of Hearing and Balance Medicine, The Affiliated Hospital of Medical School of Ningbo University, Ningbo, China

**Keywords:** antimicrobial photodynamic inactivation, photosensitizers, fullerene derivatives, antibacterial, multidrug resistance, functionalization

## Abstract

Antimicrobial photodynamic inactivation (aPDI) is a newly emerged treatment approach that can effectively address the issue of multidrug resistance resulting from the overuse of antibiotics. Fullerenes can be used as promising photosensitizers (PSs) for aPDI due to the advantages of high triplet state yields, good photostability, wide antibacterial spectrum, and permissibility of versatile functionalization. This review introduces the photodynamic activities of fullerenes and the up-to-date understanding of the antibacterial mechanisms of fullerene-based aPDI. The most recent works on the functionalization of fullerenes and the application of fullerene derivatives as PSs for aPDI are also summarized. Finally, certain remaining challenges are emphasized to provide guidance on future research directions for achieving clinical application of fullerene-based aPDI.

## Introduction

In the past few decades, the overuse of antibiotics has brought about the emergence of super bacteria with multidrug resistance, posing tremendous challenges to the treatment of bacterial infections (Prestinaci et al., 2015; Willyard, 2017; Rawson et al., 2021; Varela et al., 2021). Antimicrobial photodynamic inactivation (aPDI) is a newly emerged treatment approach that can effectively address the issues of multidrug resistance (Hu et al., 2018; Blum et al., 2020; Wang et al., 2021). Up till now, no resistance against aPDI has been reported in bacteria even after 20 successive bactericidal cycles (Giuliani et al., 2010; Hamblin, 2016, 2018; Al-Mutairi et al., 2018; Pieranski et al., 2020; Wozniak et al., 2021). aPDI starts with the injection or coating of photosensitizers (PSs) to the infected area. After the accumulation of PSs in the targeted area, irradiation with the light of specific wavelengths is applied to trigger photodynamic reactions. Under irradiation, PSs in the ground state adsorb photons and are excited into a transient singlet state, from which they enter a relatively stable triplet state by fluorescence, heat conversion, or undergoing an intersystem crossing (ISC) process (Ochsner, 1997). The two mechanisms of aPDI are as follows: (1) Type-I mechanism: PS in the triplet state produces reactive oxygen species (ROS), such as hydroxyl radicals (HO⋅), hydrogen peroxide, and superoxide anion radicals (O_2_^⋅–^) through gain or loss of electrons; and (2) Type-II mechanism: PS in the triplet state interacts with oxygen and produces singlet oxygen (^1^O_2_). ROS and ^1^O_2_ are strong oxidants that can cause disruption of the cell wall, alteration of the cell membrane structure, damage of DNA and RNA molecules, inhibition of essential enzymatic activities, denaturation of proteins, peroxidation of unsaturated lipids, leakage of intracellular substances, and disruption of the transmembrane transport system, leading to irreversible damage to the bacterial cells (Michaeli and Feitelson, 1994; Cadet et al., 2010; Allison and Moghissi, 2013; Vatansever et al., 2013; Gao et al., 2021; Rapacka-Zdonczyk et al., 2021). The Type-I mechanism does not rely on oxygen and is not limited by oxygen deprivation, while the Type-II mechanism is limited by the lack of oxygen in certain infected areas. To improve the effectiveness of aPDI treatment, the ideal PSs are required to produce high yields of ROS or ^1^O_2_ that also possess supreme bacteria-targeting abilities to minimize damage to the normal tissue.

Owing to the fact that PSs can be directly injected or coated to the infected site, aPDI will not cause damage to the entire body like antibiotics do and thus can be repetitively applied to achieve higher bactericidal efficiency while minimizing the side effects inflicted on patients (Hamblin, 2016). These advantages of aPDI have been particularly demonstrated in the treatment of dental infections, such as peri-implantitis and periodontitis (Oruba and Chomyszyn-Gajewska, 2016; Diogo et al., 2019; Tan et al., 2020, 2021; Betancourt et al., 2021; Luchian et al., 2021), and infections due to burns or damaged tissues with insufficient blood supply that are hardly available for antibiotics (Gad et al., 2004; Choudhary et al., 2009; Sperandio et al., 2010; Simonetti et al., 2021).

Among the commonly used PSs (e.g., porphyrin and its derivatives, phthalocyanine, xanthene dyes, etc.), fullerenes are promising candidates due to their high triplet state yields, good photostability, high photoactivity, and supreme ROS and ^1^O_2_ production abilities (Table 1). Fullerenes are a kind of hollow molecules composed entirely of carbon atoms with a spherical, ellipsoidal, cylindrical, or tubular shape. The condensed aromatic rings of fullerenes cause extended π-conjugation of molecular orbits (Rahmati and Mozafari, 2019; Mohammed et al., 2020). The large amounts of conjugated double bonds can effectively adsorb light in the ultraviolet (UV) and visible regions, generating ROS with high triplet yields under irradiation (Hamblin, 2018). This review introduces the photodynamic activities of fullerenes and the up-to-date understanding of the antibacterial mechanisms of fullerene-based aPDI. The most recent works on the functionalization of fullerenes and the application of fullerene derivatives as PSs for aPDI are also summarized.

**TABLE 1:** Advantages and disadvantages of commonly used PSs.

Class	PS	Advantages	Disadvantages	References
Photofrin and its derivatives	Hematoporphyrin and its derivatives (HpD)	First-generation PS	Slow metabolization; strong histotoxicity; low active constituent content; short adsorption wavelength; cause skin allergies	Gomes et al., 2018
	Verteporfin; 5-aminolevulinic acid (5-ALA); hematoporphyrin monomethyl ether (HMME); Temoporfin (m-THPC)	High ^1^O_2_ yields; high phototoxicity	Slow metabolization; low immune clearance rate; poor photostability; poor water solubility	Inoue, 2017; Gomes et al., 2018; Zhang et al., 2020
Phthalocyanine-based PS	Silicon(IV) phthalocyanine Pc4; Zinc(II) phthalocyanines	Physicochemical stability; long adsorption wavelengths (660–720 nm, with a 50-time higher adsorption at 680 nm); high immune clearance rate; low toxicity in dark	Poor water solubility; prone to agglomerate; low ^1^O_2_ yields in oxygen deprivation	Galstyan, 2021
Polycyclic quinone PS	Riboflavin; curcumin; hypericin; hypocrellin	Easy purification; high quantum yields; high phototoxicity; low toxicity in dark; good biocompatibility	Short adsorption wavelengths (400–500 nm); poor water solubility; prone to agglomerate	Hamblin, 2018; Yin and Hamblin, 2015; Rajendran, 2016
Xanthene dyes	Rhodamine; fluorescein	Good water solubility; high fluorescence quantum yields; high molar extinction coefficient; good biocompatibility; low cytotoxicity	Poor chemical stability; poor photostability; short adsorption wavelengths (∼500 nm)	Chen et al., 2012
4,4-Difluoro-4-bora-3a,4a-diaza-s-indacene (BODIPY)		Easy modification; good photophysical and photochemical stability; high fluorescence quantum yields; absorption and emission in the visible region with high molar absorption coefficients	Poor water solubility; low triplet state yield; low ^1^O_2_ yield	Durantini et al., 2018; Agazzi et al., 2019a
Transition metal complexes	Ruthenium (II); rhodium; iridium; platinum (II); gold (III)	High ROS yields; high immune clearance rate	Short excitation wavelengths; low penetration depths; poor biocompatibility	Armstrong et al., 2021; Pobłocki et al., 2021
Fullerene derivatives		High triplet state yields (∼100%); high ROS yields; good photostability; able to generate highly toxic hydroxyl radicals	Short adsorption wavelengths (400∼ 500 nm); low penetration depths; poor water solubility	Hamblin (2018); Pan et al., 2020; Heredia et al., 2022

## Advantages of Fullerene Derivatives as Photosensitizers for Antimicrobial Photodynamic Inactivation

**FIGURE 1:**
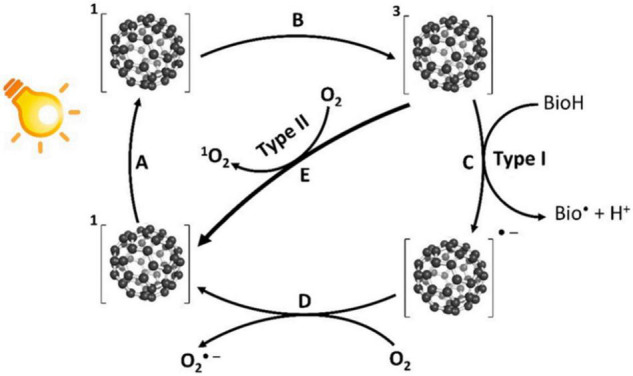
Schematic representation of the photodynamic activities of fullerenes (Heredia et al., 2022). A: Photoexcitation of fullerenes to the singlet state; B: Transition from the singlet state to the triplet state by ISC; C: Charge transfer to surrounding biomolecules to form ROS (Type-I mechanism); D: Fullerenes can further transfer an electron to O_2_ to form O_2_^⋅–^; and E: Energy transfer from the excited triplet state to O_2_ to form ^1^O_2_ (Type-II mechanism).

Fullerenes are a kind of PSs that can produce high yields of ^1^O_2_ and ROS, including O_2_^⋅–^, HO⋅, lipid hydroperoxides, and hydrogen peroxides through both Type-I and Type-II mechanisms (Yamakoshi et al., 2003; Vileno et al., 2006). Under UV or visible light irradiation, fullerenes can be excited into the transient singlet state (process A in Figure 1) and then immediately transform into the triplet state by ISC (process B in Figure 1; Guldi and Prato, 2000; Koeppe and Sariciftci, 2006). Compared with other photosensitizers, fullerenes have a higher triplet yield of nearly 100% with a longer lifetime of 50∼100 μs (Sharma et al., 2011; Huang Y.-Y. et al., 2014). Subsequent charge transfers to the surrounding biomolecules lead to the formation of ROS (Type-I mechanism as illustrated by process C in Figure 1). The remaining fullerene radicals can further transfer an electron to oxygen to form O_2_^⋅–^ (process D in Figure 1). Fullerenes in the triplet state can also transfer energy to oxygen, forming ^1^O_2_ (Type-II mechanism, as illustrated by process E in Figure 1).

Generally speaking, aPDI is more effective against Gram-positive bacteria than against Gram-negative bacteria. The cell wall of Gram-positive bacteria is a thick but porous peptidoglycan layer, while that of Gram-negative bacteria is composed of a relatively dense outer phospholipid bilayer and a thin peptidoglycan inner layer. Owing to the differences in membrane permeability and polar head size of phospholipids, which is smaller for Gram-positive bacteria (Indeglia et al., 2018), the cell wall and cell membrane of Gram-positive bacteria are easier to be penetrated by ^1^O_2_ and ROS while Gram-negative bacteria are difficult to be killed (Bertoloni et al., 1992; Kashef and Hamblin, 2017). Compared with other PSs, fullerene can produce HO⋅ free radicals with stronger toxicity that are also capable of killing Gram-negative bacteria (Huang et al., 2012). Huang L. et al. (2014) reported that fullerene-mediated PDT could treat wound infections caused by virulent Gram-negative bacterial species.

Moreover, when used in conjunction with antibiotics, fullerene-based aPDI can increase the susceptibility of multidrug-resistant bacteria to antibiotics. Wozniak et al. (2021) reported that the synergistic effect of fullerene-based aPDI and imipenem reduced the resistance of *Enterococcus faecalis* against imipenem. The use of antibiotics, including gentamycin, streptomycin, tigecycline, doxycycline, and daptomycin, accompanied by fullerene-based aPDI exhibited a more pronounced inhibition of multidrug-resistant *Enterococcus faecium* than when these antibiotics were used alone. The resensitization of multidrug-resistant bacteria to antibiotics could be attributed to the aPDI-induced increase in membrane permeability (Wozniak et al., 2021).

Other advantages of fullerenes as a PS are as follows: (1) High resistance to photobleaching. Many traditional PSs completely lose their photoactivity after relatively low total energy doses (<10 J/cm^2^), while fullerenes can retain photoactivity even after very high energy doses (>1,000 J/cm^2^) (Hamblin, 2018). (2) Abundance of double bonds on the surface that allows versatile functionalization. (3) Oxygen-independent photokilling through Type-I mechanism. These combined advantages make fullerenes supreme PSs with promising prospects of application in aPDI.

## Antibacterial Mechanisms of Fullerene-Based Antimicrobial Photodynamic Inactivation

**FIGURE 2:**
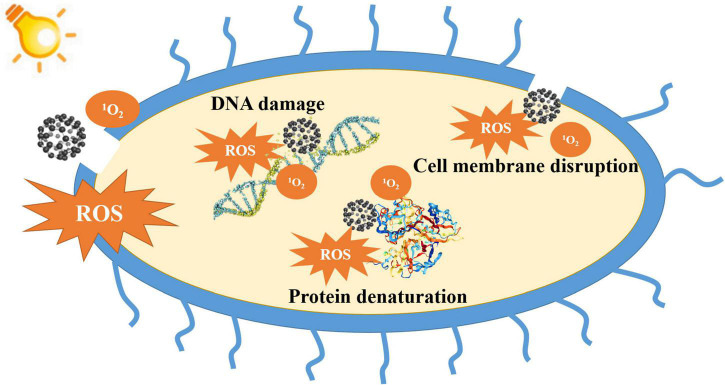
Schematic representation of the photodynamic antibacterial mechanisms of fullerenes.

To deepen the understanding of fullerene-based aPDI, researchers have strived to explore the underlying photodynamic antibacterial mechanisms of fullerenes. Up till now, three major mechanisms have been proposed: disruption of the cell membrane, denaturation of proteins, and induction of DNA damage (Figure 2).

Fullerene-based aPDI can lead to the peroxidation of lipids, causing bacterial cell membrane disruption, cytoplasm leakage, and eventually cell death (Sayes et al., 2004). Fang et al. (2007) found that the photodynamic reactions of fullerene changed the composition of the bacterial cell membrane in a strain-dependent manner. For Gram-negative bacterium *Pseudomonas putida*, aPDI decreased the proportions of unsaturated fatty acids while increasing that of cyclopropane fatty acids. For Gram-positive *Bacillus subtilis*, aPDI increased the proportions of monounsaturated fatty acids and membrane fluidity. Grinholc et al. (2015) also reported that photodynamic reactions of fullerene compromised the structural integrity of the cell membrane of *Staphylococcus aureus*, indicating that the bacterial cell membrane is a major target for fullerene-based aPDI. In addition, the inherent hydrophobic structure of fullerenes can cause mechanical disruption of the cell membrane. Even at low concentrations, fullerenes can adhere to and insert into the cell membrane, leading to the change in membrane permeability and leakage of intracellular substances, eventually causing bacterial death (Zhang et al., 2021). Furthermore, fullerenes can cause protein denaturation under irradiation (Radic et al., 2014). Nakagawa et al. (2014) reported that fullerenols induced necrocytosis and apoptosis of bacteria by causing protein denaturation through oxidation of cysteine.

Fullerenes have also been reported to bind DNA molecules after entering microbial cells and change the structures and biochemical functions of DNA, inducing oxidative stress and cellular respiration interruption (Lyon and Alvarez, 2008; Nishizawa et al., 2009; An and Jin, 2011). Moreover, fullerenes are prone to adhere to DNA chains due to their high affinity to guanine, causing damage to DNA and eventually bacterial death (Zhao et al., 2005). An and Jin (2015) found that fullerenes bound to nucleic acids and disrupted cell reproduction and regeneration, leading to apoptotic responses of bacteria. Additionally, the ^1^O_2_ and O_2_^⋅–^ generated by fullerene can cause photocleavage of DNA chains (Ikeda et al., 2007). Iwamoto and Yamakoshi (2006) reported DNA cleavage induced by C_60_–N-vinylpyrrolidone copolymer. It has also been reported that the ^1^O_2_ produced by fullerene induced DNA mutation in *Salmonella* strains under visible light irradiation (Sera et al., 1996).

## Application of Fullerene Derivatives in Antimicrobial Photodynamic Inactivation

Despite the many advantages of fullerene as a PS, there exist certain drawbacks, limiting the application of fullerene in aPDI. First of all, the inherent hydrophobicity and poor water solubility of fullerene make the injection of fullerene into the target area virtually impossible. Fullerene nanoparticles are also prone to agglomerate in a liquid environment, adversely affecting their photoactivity (Mizuno et al., 2011). Second, the ROS and ^1^O_2_ generated by the photodynamic reactions of fullerene may also inflict damage on normal tissue. Lack of ability to target bacterial cells leads to increased cytotoxicity, limiting the clinical use of fullerene-based aPDI. Furthermore, the adsorption range of fullerene is limited to the green, blue, or UV region (Heredia et al., 2022). The applicability of fullerene-based aPDI in deep areas of the body is limited by the poor penetration depth of light of short wavelengths. Nevertheless, the abundant double bonds of fullerene allow surface grafting of various functional groups for increased water solubility, improved targeting ability, and broadened adsorption range. In recent years, research attention is being increasingly focused on the synthesis and development of functionalized fullerene derivatives for application in aPDI (Zhang et al., 2019; Pan et al., 2020; Pochkaeva et al., 2020; Heredia et al., 2022).

### Cationic Fullerene Derivatives

The ideal PSs for aPDI shall selectively attack microbes without damaging mammalian cells. It was found that bacterial cell surfaces are more negatively charged compared to mammalian cell surfaces. Therefore, grafting cationic functional groups on the surface of fullerene not only improves water solubility, but also facilitates targeted attacks on bacteria by rapidly binding to the anionic residues on the bacterial cell wall (Merchat et al., 1996; Mashino et al., 2003; Hamblin, 2016). Additionally, the cellular uptake of cationic fullerene derivatives by bacteria is significantly greater than that by mammalian cells (Soncin et al., 2002), making cationic fullerene derivatives perfect PSs for aPDI. Based on the type of cationic functional groups, cationic fullerene derivatives can be classified into fullerene-pyrrolidine derivatives and fullerene-cyclopropane derivatives.

#### Fullerene-Pyrrolidine Derivatives

**FIGURE 3:**
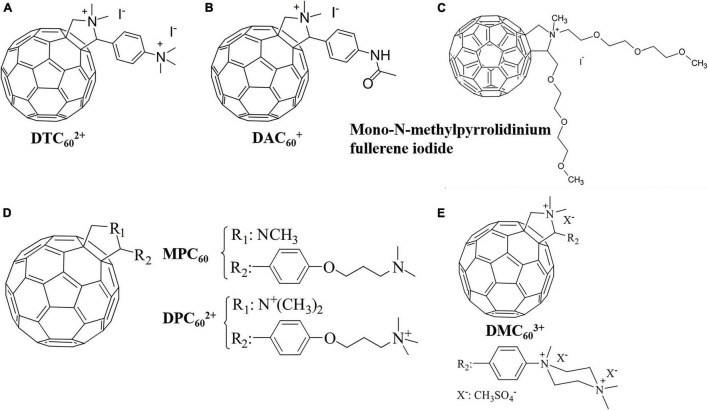
Molecular structures of the fullerene-pyrrolidine derivatives. **(A)** DTC_60_^2+^. **(B)** DAC_60_^+^ (Spesia et al., 2008). **(C)** Mono-N-methylpyrrolidinium fullerene iodide (Grinholc et al., 2015). **(D)** MPC_60_ and DPC_60_^2+^ (Agazzi et al., 2015). **(E)** DMC_60_^3+^ (Agazzi et al., 2021).

Pyrrolidination of fullerene is one of the main approaches to prepare cationic fullerene derivatives. (Huang Y.-Y. et al., 2014) grafted electroneutral dienol groups and positively charged pyrrolidinium groups on fullerene and compared the antibacterial effectiveness and spectrum of the two derivatives. It was found that the neutral dienol-functionalized fullerene exhibited only modest antibacterial activity against Gram-positive *Staphylococcus aureus*, while the cationic pyrrolidinium-functionalized fullerene effectively induced light-mediated killing of Gram-positive *S. aureus*, Gram-negative *Escherichia coli* and *Pseudomonas aeruginosa*, and fungal yeast *Candida albicans* by inhibiting oxygen uptake. Spesia et al. (2008) investigated the relationship between the number of cationic charges of fullerene-pyrrolidine derivatives and aPDI effectiveness against *E. coli* suspensions. After 30 min of irradiation, the dicationic fullerene derivative DTC_60_^2+^ (Figure 3A) and the monocationic fullerene derivative DTC_60_^+^ (Figure 3B) decreased the viability of *E. coli* by 99.97 and 96.8%, respectively, while the non-charged fullerene derivative MAC_60_ showed negligible effect on *E. coli*. These results indicate that the effectiveness of aPDI is positively correlated to the number of charges on cationic fullerene derivatives.

Grinholc et al. (2015) prepared a mono-N-methylpyrrolidinium fullerene iodide (Figure 3C) with supreme bactericidal effect on *E. coli*, *P. aeruginosa*, *S. aureus*, and *C. albicans* under white light irradiation. The bacterial inactivation observed was attributed to cell membrane disruption induced by ^1^O_2_ and O_2_^⋅–^ instead of DNA cleavage. *In vivo* studies showed that the as-prepared fullerene derivative was effective in treating mice infected with *S. aureus* (Grinholc et al., 2015). Agazzi et al. (2015) synthesized a novel fulleropyrrolidine C_60_ derivative (MPC_60_) containing an aliphatic chain with an amino group at the end. By exhaustive methylation of the amino group, a dicationic fulleropyrrolidinium (DPC_60_^2+^) was further synthesized (Figure 3D). Although MPC_60_ generated higher yields of ^1^O_2_ than DPC_60_^2+^, higher photoinactivation of *S. aureus* was reported for the dicationic DPC_60_^2+^. After incubation with 0.5 μM fullerene and irradiation for 30 min, MPC_60_ and DPC_60_^2+^ decreased the cell viability by 4.4 and 5.0 log, respectively. Oxygen was found to be essential for effective aPDI by DPC_60_^2+^, and ^1^O_2_ was detected in *S. aureus* cells. After the addition of NADH (which is able to quench ^1^O_2_) into the bacterial suspension, a weakened aPDI performance was observed, indicating that DPC_60_^2+^ killed *S. aureus* mainly through Type-II mechanism (Gsponer et al., 2016). In 2021, the same research group prepared an amphiphilic tricationic fullerene derivative DMC_60_^3+^ (Figure 3E) with strong UV adsorption that effectively inhibited the growth of *E. coli* and *S. aureus* through the synergistic effect of Type-I and Type-II mechanisms (Agazzi et al., 2021).

Apart from the number of cationic charges, the position of charges may also affect the aPDI effectiveness of fullerene-pyrrolidine derivatives. Mizuno et al. (2011) found that distributing cationic charges around the fullerene cage can minimize the tendency of fullerene to aggregate and enhance their aPDI effectiveness. Palacios et al. (2021) prepared four fullerene-pyrrolidine derivatives and found that compounds with the highest dipole moments exhibited a stronger inactivation effect against methicillin-resistant *E. coli* and *S. aureus*.

#### Fullerene-Cyclopropane Derivatives

**FIGURE 4:**
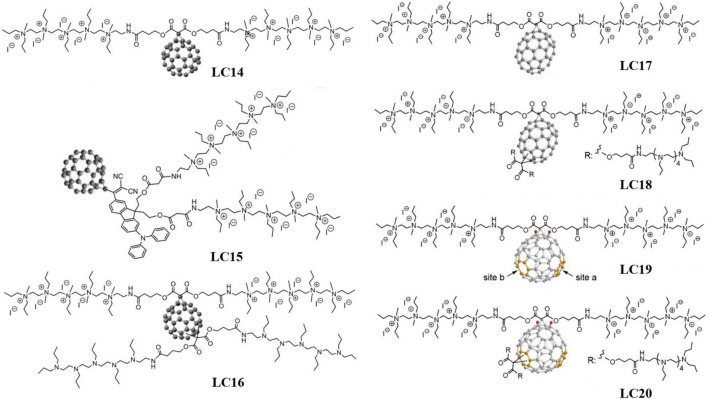
Molecular structures of the fullerene-cyclopropane derivatives, LC14–LC20 (Sperandio et al., 2013; Yin et al., 2015).

Cyclopropanation is another common approach to prepare cationic fullerene derivatives. Fullerene-cyclopropane derivatives can effectively target Gram-negative bacteria due to their rich abundance of quaternary ammonium cations. When irradiated with UV (wavelength 320∼420 nm) or white light, fullerene-cyclopropane derivatives can generate both ^1^O_2_ and ROS (predominantly HO⋅) with bactericidal potencies against multiple drug-resistant bacterial species. Yin et al. (2015) synthesized three fullerene-cyclopropane derivatives as PSs for aPDI, C_60_[>M(C_3_N_6_^+^C_3_)_2_]-(I^–^)_10_ (LC14), C_60_[>CPAF-(MN_6_^+^C_3_)_2_]-(I^–^)_10_ (LC15), and C_60_[>M(C_3_N_6_^+^C_3_)_2_][>M(C_3_N_6_C_3_)_2_]-(I^–^)_10_ (LC16), and their molecular structures are shown in Figure 4. Zhang et al. (2015) investigated the ROS-generating ability of these three fullerene derivatives and their inhibition effectiveness against methicillin-resistant *S. aureus*, *E. coli*, and *C. albicans*. LC15 showed the best broad-spectrum antibacterial properties, followed by LC16 and LC14 in sequence. Owing to the lack of electron-donating groups, LC14 could only produce ^1^O_2_ via a Type-II mechanism that is only effective against Gram-positive bacteria. Nevertheless, ^1^O_2_ cannot effectively kill Gram-negative bacteria due to the difficulty in penetrating their cell wall. LC15, which produces HO⋅, was more effective against Gram-negative bacteria. Wang et al. (2013) prepared a water-soluble decacationic fullerene derivative C_60_[>M(C_3_N_6_^+^C_3_)_2_]-(I^–^)_10_ capable of producing both ^1^O_2_ and HO⋅ under UV and white light irradiation. The 10 quaternary ammonium cations per C_60_ provided the compound with excellent bactericidal ability against pathogenic Gram-positive and Gram-negative bacteria. Wang et al. (2012) compared the bactericidal effect of decacationic C_60_ and C_70_ derivatives C_60_[>M(C_3_N_6_^+^C_3_)_2_] and C_70_[>M(C_3_N_6_^+^C_3_)_2_] (LC17). Compared with C_60_[>M(C_3_N_6_^+^C_3_)_2_], C_70_[>M(C_3_N_6_^+^C_3_)_2_] was more effective against Gram-negative bacteria due to its ability to produce HO⋅ via the Type-I mechanism. Based on LC17, Sperandio et al. (2013) added a deca-tertiary ethyleneamino-chain as an electron source and prepared C_70_[>M(C_3_N_6_^+^C_3_)_2_][M(C_3_N_6_C_3_)_2_] (LC18). Huang L. et al. (2014) reported excellent effectiveness of LC18 in treating mice with third-degree burns infected by Gram-negative bacteria. The 10 tertiary amine groups of LC18 (with each tertiary amine group providing one electron for the C_70_ cage) enable the production of HO⋅ under UV light irradiation. Sperandio et al. (2013) further replaced the C_70_ of LC17 and LC18 with C_84_O_2_ and prepared C_84_O_2_[>M(C_3_N_6_^+^C_3_)_2_] (LC19) and C_84_O_2_[>M(C_3_N_6_^+^C_3_)_2_][M(C_3_N_6_C_3_)_2_] (LC20). LC19 was found to exhibit stronger phototoxicity when irradiated with light of longer wavelengths (615∼645 nm), while LC20 exhibited stronger phototoxicity at short wavelengths (350–420 nm).

### Fullerenols

Fullerenols are a family of polyhydroxylated fullerene that can be used as PSs for aPDI and are prepared by chemical functionalization of fullerene using hydrogen peroxide. Pickering and Wiesner (2005) first proved the ability of fullerenols to simultaneously produce ^1^O_2_ and O_2_^⋅–^ under UV or polychromatic light irradiation. Fullerenols were found to exhibit 2∼3 times greater light adsorption in the wavelength range of 300∼400 nm than in the visible range, accompanied by an order of magnitude greater ROS generation rate. Compared with C_60_ nanoparticles, fullerenols possess stronger abilities to bind bacterial DNA and induce DNA mutations (An and Jin, 2011). Aoshima et al. (2009) reported that fullerenols C_60_(OH)_12_, C_60_(OH)_36_.8H_2_O, and C_60_(OH)_44_.8H_2_O significantly inhibited the growth of *C. albicans*, *Malassezia furfur*, and *Propionibacterium acnes*. Furthermore, C_60_(OH)_44_.8H_2_O was found to inhibit the growth of *E. coli*, *S. aureus*, *Staphylococcus epidermidis*, and *Bacillus* sp. Brunet et al. (2009) found that the photodynamic activities of fullerenols were dependent on the solution in which they were suspended. When suspended in ultrapure water, fullerenols generated abundant ^1^O_2_, while when suspended in minimal Davis microbial growth medium, fullerenols generated both ^1^O_2_ and O_2_^⋅–^. However, when suspended in tetrahydrofuran, fullerenols exhibited no photoactivity. Kuo et al. (2020) reported massive production of ROS and complete elimination of methicillin-resistant *S. aureus* by water-soluble fullerenol C_60_(OH)_30_ under two-photon excitation in the near-infrared region (760 nm).

Fullerenols can also be used in conjunction with other antibacterial agents for enhanced aPDI performances. Krishna found that the adsorption of polyhydroxyl fullerene on the surface of TiO_2_ nanoparticles increased the photocatalytic performance of TiO_2_ nanoparticles by 70% through scavenging of photo-generated electrons. The combination of fullerene and TiO_2_ achieved a 2-fold higher killing rate against *E. coli* due to increased yields of HO⋅ (Krishna et al., 2008). Bai et al. (2012) synthesized a nanocomposite of polyhydroxy fullerene and TiO_2_ that can effectively kill microbes, such as *Aspergillus niger*, under visible light irradiation. The spore inactivation rate of this nanocomposite was 3∼4 times greater than that of pure TiO_2_. Wan et al. (2021) used fullerenols in conjunction with ultra-small copper nanoparticles to achieve synergistic antibacterial and antioxidative effects. The addition of fullerenols reduced the usage of copper nanoparticles, alleviating the copper-induced environmental harm. Ansari et al. (2022) loaded the surface of fullerenols with an antibacterial drug sulfasalazine and found that the addition of fullerenols decreased the minimum inhibitory concentration (MIC) of sulfasalazine against *Bacillus cereus*, *Proteus mirabilis*, and *Salmonella typhimurium*, but not against *E. coli* or *S. aureus*.

### Fullerene Derivatives With Light-Harvesting Antenna

**FIGURE 5:**
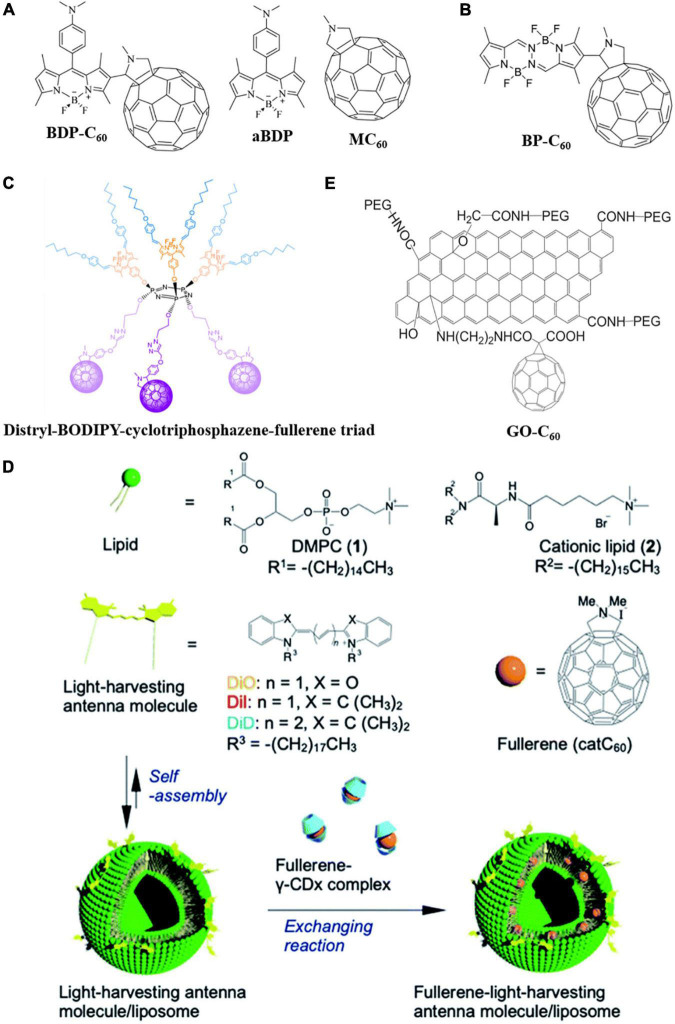
Molecular structures of the fullerene derivatives with a light-harvesting antenna. **(A)** BDP-C_60_, aBDP, and MC_60_ (Agazzi et al., 2019b). **(B)** BP-C_60_ (Lopez et al., 2021). **(C)** Distryl-BODIPY-cyclotriphosphazene-fullerene triad (Sarikaya et al., 2019). **(D)** Schematic diagram of LMIcatC_60_–light-harvesting antenna molecules (Kawasaki et al., 2020). **(E)** GO-C_60_ (Li et al., 2017).

Despite the supreme ^1^O_2_ and ROS-generating ability, the applicability of fullerenes as a PS for aPDI in deep areas of the body is limited due to their short adsorption wavelengths (usually green, blue, or UV light which is hard to penetrate deep into the body) (Agazzi et al., 2015; Sobotta et al., 2019). Thus, covalently grafting light-harvesting agents, such as chromophores, on the surface of fullerene to broaden its adsorption spectrum is a desirable strategy to improve the aPDI effectiveness of fullerene in deep areas of the body. 4,4-Difluoro-4-bora-3a,4a-diaza-s-indacene (BODIPY) has the advantages of good photostability, low toxicity, high adsorption, and fluorescence emission in the visible range, and thus can be covalently grafted on the surface of fullerene as a “light-harvesting antenna” to increase the generation of excited triplet state and produce ^1^O_2_ through the Type-II mechanism (Wei et al., 2017; Durantini et al., 2018; Agazzi et al., 2019a,b). Agazzi et al. (2019b) grafted dimethylaminophenyl-BODIPY (aBDP) on the surface of N-methylfulleropyrrolidine (MC_60_) to synthesize a novel C_60_-BODIPY dyad (BDP-C_60_) (Figure 5A). The aBDP acted as a “light-harvesting antenna” and singlet energy or electron donor, while C_60_ served as singlet energy or electron acceptor to produce triplet state C_60_. Grafting of BODIPY red-shifted the adsorption peak from 415 to 515 nm, even longer than that of pure BODIPY (502 nm). Additionally, the ^1^O_2_ yield was found to be correlated with solvent polarity. In the toluene solution with strong polarity, ^1^O_2_ yield was high; while in the dimethylformamide solution with weak polarity, ^1^O_2_ yield was relatively low. The presence of BODIPY also increased the ROS yield of fullerene. The dyad exhibited excellent aPDI effectiveness at physiological pH, decreasing the viability of *S. aureus* by 99.97% and *E. coli* by 99.98% within 15 and 30 min of green light irradiation, respectively. Covalently binding a bis(difluoroboron)-1,2-bis((1H-pyrrol-2-yl)methylene)hydrazine (BOPHY) fluorophore unit to N-methylfulleropyrrolidine, Lopez et al. (2021) synthesized a BOPHY-C_60_ (BP-C_60_) dyad (Figure 5B) in which the BOPHY “antenna” not only improved light adsorption in the visible range, but also enhanced the photodynamic performance of the fullerene moiety. Under irradiation, the dyad effectively inactivated *S. aureus* by generating ROS through both Type-I and Type-II mechanisms in the presence of oxygen. However, this dyad did not exhibit a significant aPDI effect against *E. coli*. Sarikaya et al. (2019) synthesized a distryl-BODIPY-cyclotriphosphazene-fullerene triad (Figure 5C) with light adsorption and emission in the near-IR range, potentially extending the application of fullerene in aPDI. Agazzi et al. (2020) prepared a diketopyrrolopyrrole-C_60_ conjugate in which diketopyrrolopyrrole dyes acted as a light-harvesting antenna. The conjugate exhibited a high level of ROS generation and bactericidal effect against *S. aureus* under green light irradiation.

Kawasaki et al. (2020) combined fullerene with light-harvesting agents using liposomes as a platform. Light-harvesting antenna molecules [DPMC and dyes, such as 1,1′-dioctadecyl-3,3,3′,3′-tetramethylindocarbocyanine perchlorate (DiI), λ_max_ = 549 nm, 1,1′-dioctadecyl-3,3,3′,3′-tetramethylindodicarbocyanine (DiD), λ_max_ = 644 nm, and 3,3′-dioctadecyloxacarbocyanine perchlorate (DiO), λ_max_ = 484 nm] were first grafted on liposome surfaces, followed by the self-assembly of fullerene-cyclodextrin derivatives into liposomes to form LMIcatC60–light-harvesting antenna molecules (Figure 5D). Photoactivation of the antenna molecules efficiently transferred energy to fullerenes, eliminating bacteria without causing harm to erythrocytes. Gotfredsen et al. (2017) prepared a class of thieno-fused subporphyrazines with broad adsorption spectra and red-shifted adsorption limits as light-harvesting agents. These molecules are easy to co-crystallize with C_60_ and thus could be used as antennas to improve the aPDI effectiveness of fullerene. Li et al. (2017) covalently grafted graphene oxide (GO) as a light-harvesting antenna on fullerene to produce a PS for aPDI (Figure 5E). Taking advantage of GO’s strong adsorption in the near-IR range, the adsorption spectrum of GO-C_60_ was significantly broadened to enable photodynamic treatment in deep areas of the body.

### Fullerene Derivatives Modified With Amino Acids and Peptides

**FIGURE 6:**
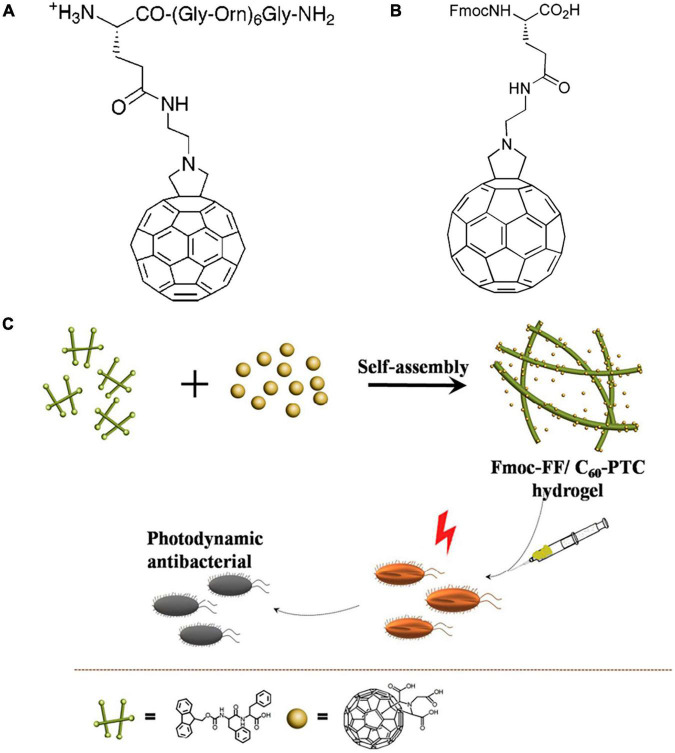
**(A)** The molecular structure of C_60_-functionalized amino acid with N-Fmoc-L-glutamic acidr-tert-butyl ester (Pellarini et al., 2001). **(B)** The molecular structure of Fgu (Pantarotto et al., 2002). **(C)** Schematic diagram of Fmoc-FF/C_60_-PTC hydrogel (Zhang et al., 2018).

Fullerene derivatives can be modified with amino acids and peptides through nucleophilic addition reactions (Yang et al., 2014; Pochkaeva et al., 2020). Kornev et al. (2012) prepared pentakisamino derivatives of C_60_ using C_60_Cl_6_ as a precursor with strong antibacterial activities against *E. coli* and *B. subtilis*. Antibacterial peptides are a family of novel antibacterial agents that kill bacteria by inducing cell membrane rupture and cellular content leakage via electrostatic interactions with the cell membrane (Raheem and Straus, 2019; Yan et al., 2021). Nevertheless, the bactericidal performance of antibacterial peptides against Gram-negative bacteria is limited by the relatively dense outer cell membrane. Conjugation of fullerene and antibacterial peptides can greatly overcome this limitation. Generally, there are two common strategies to combine fullerene with peptides: (1) Covalently grafting pre-formed peptides on fullerene; and (2) Introducing the fullerene fragment as a special amino acid into the peptide sequence (Pochkaeva et al., 2020). Using the first approach, Pellarini et al. (2001) prepared a C_60_-functionalized amino acid by condensing a fullerene derivative containing a free amino group with N-Fmoc-L-glutamic acidr-tert-butyl ester (Figure 6A). The unmodified peptides showed no antibacterial activity against *E. coli* or *S. aureus*. Conjugation of fullerene significantly enhanced the antibacterial performance, with a MIC of 8 and 64 μM against *S. aureus* and *E. coli*, respectively. Pantarotto et al. (2002) adopted the second strategy and introduced a fulleropyrrolidino-glutamic acid residue (Fgu) into the peptide sequence (H-Gly-(Nle)_2_-Gln-Orn-Nle-Gly-(Orn)_2_-Nle-(Orn)_2_-Nle-Gly-(Orn)_2_-Nle-Gly-Tyr-NH_2_) using solid-phase synthesis and investigated the influence of the position of Fgu on aPDI performance (Figure 6B). Compared with other cationic antimicrobial peptides, the Fgu-functionalized peptides exhibited significantly higher antibacterial activities against *S. aureus*. The introduction of Fgu at the N terminus of the peptide achieved better antibacterial effectiveness than in the middle of the peptide. Zhang et al. (2018) fabricated a hybrid hydrogel containing C_60_ pyrrolidine tris-acid (C_60_-PTC) and amphiphilic small peptides (Fmoc-FF) (Figure 6C). The hybrid hydrogel effectively inhibited *S. aureus* growth by generating ROS and promoted wound healing.

### Fullerene as Additives to Other Materials

**FIGURE 7:**
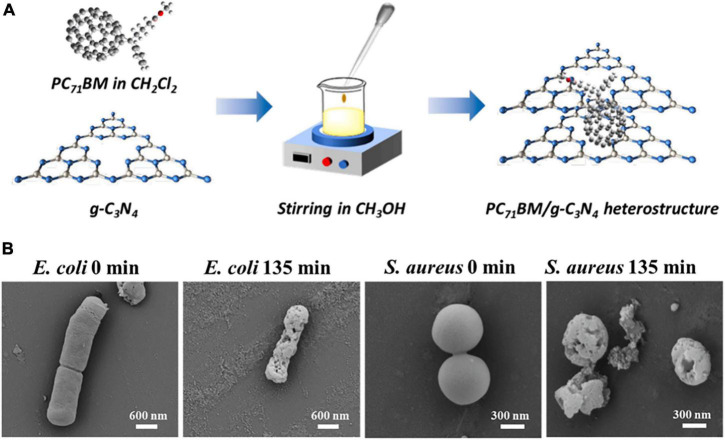
**(A)** Schematic diagram of the fabrication of PC_71_BM/g-C_3_N_4_ photocatalysts. **(B)** SEM images of *E. coli* and *S. aureus* before and after irradiation for 135 min with PC_71_BM/g-C_3_N_4_ (Liu et al., 2022).

Fullerenes can also be used as additives to other materials for optimized antibacterial performance. Gudkov et al. (2021) added fullerene as an additive to borosiloxane polymers and observed noticeable ROS production and bacterial inhibition under visible light irradiation. The ROS yield of the composite increased with the increase of the amount of fullerene added. A reliable indicator of oxidative damage to DNA by ROS is the levels of long-lived active forms of proteins (LRPS) and 8-oxoguanine (8-OH-Gua; Matsuda et al., 2011). It was observed that the composite induced a significant increase in LRPS and 8-OH-Gua levels, indicating that the ROS produced by fullerene caused oxidative damage to bacterial DNA and cell death. Liu et al. (2022) introduced a self-assembled fullerene derivative [6,6]-phenyl-C_71_-butyric acid methyl ester (PC_71_BM) to the surface of g-C_3_N_4_, a polymer-like organic photocatalytic semiconductor (Figure 7A) to prepare heterostructures for water purification. The presence of PC_71_BM extended the adsorption range of g-C_3_N_4_ from 450 to 650 nm and generated abundant ROS under irradiation. The heterostructures achieved a bactericidal rate of over 99.7% against *S. aureus* and *E. coli*, and effectively inactivated their biofilms after prolonged irradiation (Figure 7B).

## Conclusion and Perspectives

Overuse of antibiotics has brought about the issue of multidrug resistance of bacteria. In the past decades, various novel antibacterial strategies have been proposed as potential alternatives to antibiotics (Alavi and Rai, 2021; Fazeli-Nasab et al., 2021; Khodadadi et al., 2021; Ahmadi, 2022; Alavi et al., 2022a,b). As a newly emerged antibacterial strategy, aPDI can effectively address the issue of drug resistance and thus has promising prospects of application in treating bacterial infections. Fullerene can be used as a PS for aPDI for its high ^1^O_2_ and ROS yields and bactericidal activities against both Gram-positive and Gram-negative bacteria. This review summarizes the up-to-date studies on the application of fullerene derivatives in aPDI, including fullerene-pyrrolidine derivatives, fullerene-cyclopropane derivatives, fullerenols, fullerene derivatives with light-harvesting antenna, fullerene derivatives modified with amino acids and peptides, and fullerenes as additives to other materials.

Grafting cationic functional groups on the surface of fullerenes is an effective strategy to improve their ability to target negatively charged bacterial cells. Nevertheless, it is worth noting that surface modification may affect the photophysical and photochemical properties of fullerene, resulting in decreased triplet state yield and ROS generation. Moreover, although fullerene-cyclopropane derivatives have a greater number of cationic charges, they are also of higher molecular weights and thus are more difficult to penetrate into bacteria. Therefore, it is desirable to find a balance between the number of cationic charges and molecular weight for optimized bactericidal efficacy.

The adsorption wavelengths of fullerenes are limited to the green, blue, and UV regions, limiting their use in deep areas of the body. Although grafting light-harvesting antennas is a potent strategy to broaden the adsorption range of fullerenes, application of this strategy in aPDI is still rare and remains to be explored. Conjugation of fullerenes can also increase the susceptibility of bacteria to antibacterial peptides. However, the binding site of fullerenes has a considerable influence on the bactericidal efficacy of antibacterial peptides, which is worthwhile to be extensively investigated. Additionally, fullerenes also possess ROS clearance ability in the dark, which is beneficial to the proliferation of normal cells and tissue growth. This advantage makes fullerenes promising candidates as additives to wound dressings and dental implants with both antibacterial and wound healing promotion abilities.

Furthermore, the shape, structure, and performance of fullerene derivatives are closely related to preparation methods. Distinct antibacterial performances have been reported for the same fullerene derivatives prepared with different methods, and for those commercially manufactured and prepared in the laboratory (Indeglia et al., 2018). Therefore, it is needed to further investigate the relationships between preparation methods, molecular structures, and antibacterial performance to optimize and standardize the preparation of fullerene derivatives. Finally, more attention needs to be paid to the histotoxicity resulting from the accumulation of fullerene nanoparticles in organs, such as the liver and the spleen (Shipkowski et al., 2019). To address this issue, the interactions between fullerene nanoparticles and serum proteins need to be disclosed, and the influence of such interactions on their *in vivo* destiny needs to be investigated.

## Author Contributions

WH, GS, JM, and LS: writing – original draft preparation. WH and SW: writing – review and editing. XZ, WH, and YZ: funding acquisition. XZ and YZ: resources. All authors contributed to the article and approved the submitted version.

## Conflict of Interest

The authors declare that the research was conducted in the absence of any commercial or financial relationships that could be construed as a potential conflict of interest.

## Publisher’s Note

All claims expressed in this article are solely those of the authors and do not necessarily represent those of their affiliated organizations, or those of the publisher, the editors and the reviewers. Any product that may be evaluated in this article, or claim that may be made by its manufacturer, is not guaranteed or endorsed by the publisher.
